# In Utero Programming of Testicular Cancer

**DOI:** 10.3390/jdb9030035

**Published:** 2021-08-29

**Authors:** David Elad, Ariel J. Jaffa, Dan Grisaru, Ilan Leibovitch

**Affiliations:** 1 Department of Biomedical Engineering, Faculty of Engineering, Tel-Aviv University, Tel-Aviv 6997801, Israel; 2 Department of Obstetrics and Gynecology, Lis Maternity Hospital, Tel-Aviv Medical Center, Tel-Aviv 6423906, Israel; drajaffa@gmail.com; 3 Sackler Faculty of Medicine, Tel Aviv University, Tel Aviv 6997801, Israel; 4 Department of Gynecological Oncology, Lis Maternity Hospital, Tel-Aviv Medical Center, Tel-Aviv 6423906, Israel; grisaro@tauex.tau.ac.il; 5 Department of Urology, Meir Medical Center, Kfar Saba 4428164, Israel; ilan.leibo@gmail.com

**Keywords:** testicular germ cell cancer (TGCC), peri-conception, early life factors, nutrition programming, fetal development, developmental biology

## Abstract

It is well established that the intrauterine biological environment plays important roles in fetal development. In this review, we re-visit the hypothesis that testicular germ cell cancer (TGCC), especially in adolescents and young adults, has been programmed in utero. The origin for extreme in utero environments is mostly maternal driven and may be due to nutritional, physical and psychological stressful conditions that alter the optimal molecular and biophysical in utero environments. Moreover, precursors for TGCC may originate as early as during fertilization or implantation of the blastocyst. Further investigations of human developmental biology, both in vivo and in vitro, are needed in order to establish better understanding of in utero programming of future wellbeing or diseases.

## 1. Introduction

The phrases fetus programming, in utero programming or early-life factors have been recently suggested to describe irregular, unknown maternal events before and during pregnancy that may lead to developmental alterations responsible for short and long term pathologies and diseases, and especially cancer. In the absence of convincing scientific evidence in humans for either normal factors of fetal development or the etiology of diseases, the National Cancer Institute (NCI) organized in 2011 a Workshop on “Early-life Events and Cancer” [1]. In 2018, the National Health Institute (NIH) opened a funding opportunity (PA-18-532) titled “Early-life Factors and Cancer Development Later in Life” to study the role of early-life factors in cancer development later in life, including maternal-paternal, in utero, birth and infancy.

The existing knowledge is mostly based on epidemiology research, in which retrospective information was used to determine cancer risk factors after delivery or in adulthood. Very little, if any, was focused on early-life factors. It is surprising that the metrology for healthy newborns is still based on correlations between overall morphological factors such as weight and length at birth or similar non-relevant factors for the etiology of pathologies during fetal development [2]. In this respect, the medical sciences still lack knowledge and technologies for reliable and reproducible cell metrology concerning the mechanisms of developmental biology and functional performance [3].

It is well established that the intrauterine environment plays important roles in fetal development [4]. During pregnancy, maternal-fetal nutrition interaction is a two-way delicate process that directly affects fetal development. For over 140 years, it has been known that untreated diabetes interferes with fertility, and during pregnancy it largely affects the outcome [5]. In more general terms, unbalanced nutrition, whether under- or over-nutrition, adversely affects the developmental biology of fetal cells. In more mechanistic terminology, it was suggested that inappropriate maternal-mediated fuel may induce alterations in fetal cell development with subsequent teratogenesis in behavioral, anthropometric and metabolic functions [6]. In addition, animal experiments revealed that some tissue (e.g., brain, adipose, muscle) complete their development during early childhood and stop renewal [7]. Thus, if things go wrong in utero, it is impossible to stop the pathway for disease development [8]. Only recently have new reprogramming technologies emerged with the idea of bringing aging and pathological tissue back in time to allow for regeneration [9].

## 2. Testicular Germ Cell Cancer

Testicular cancer is relatively rare in the global population, but accounts for up to 2% of male malignancies [10]. The incidence rate in North America and Europe is between 5 to 8.7 cases per 100,000 men per year, but has shown constant growth in the past decades [11,12]. In the 1970s, malignant testicular cancer was established as testicular germ cell cancer (TGCC) [13]. In brief [14], TGCCs are most likely derived from primitive germ cells during early embryogenesis and become visible during young adulthood at a very low annual frequency [15]. These cancers are classified into seminoma and non-seminoma that can be either localized or metastatic. In most of the non-seminoma TGCC patients, the tumor may be composed of pure or mixed histological elements of embryonal carcinoma, teratoma, yolk sac tumor and choriocarcinoma.

In 2016, the World Health Organization (WHO) revised the clinical classification of testicular tumors and unified the nomenclature of the TGCC precursor to be germ cell neoplasia in situ (GCNIS) [16]. In this new classification, new concepts of TGCC with delayed maturation beyond childhood, and thus, immature germ cells were confused with GCNIS. The fundamental change was the subdivision of TGCC into pathologies derived from GCNIS and those not derived from GCNIS. Detailed descriptions are given in Ulbright [16]. In the context of this article, it should be noted that among the GCNIS-derived tumors there are three forms of nonchoriocarcinomatous trophoblastic tumors, including the placental site trophoblastic tumor. It is a very rare type of testicular cancer that usually appears along with TGCC [17,18], but nevertheless, it is a gestational trophoblastic neoplasm that originates from the placental implantation site [19], and thus, the precursors were most likely imprinted in utero.

## 3. Testicular Cancer in Young Adults

Testicular cancer is the most common malignant cancer in adolescents and young adults under 40 years, and comprises about 14% of adolescent malignancies [10,20,21]. The peak incidence of testicular cancer in the USA is around 14 cases per 100,000 persons for the age group of 25 to 34 years with non-Hispanic whites and Hispanics having the highest rates [20]. Current studies and future forecasts show a continuous increase in testicular cancer incidence, especially in Hispanic men [22]. The challenge of treatment for this group is not only survival, but also includes issues such as secondary malignancy, fertility and psychosocial relationships, which require more in-depth research [21]. Accordingly, we recall two examples that may provide more perspectives for future investigation. The first example comprises two brothers in a set of quadruplets that were born in Lithuania in 1969 and diagnosed with testicular cancer in their early 20s during military service [23]. Both were diagnosed with testicular teratoblastoma (i.e., a tumor containing embryonic tissue), and died 11 and 18 months, respectively, post orchiectomy and combination chemotherapy.

The second example includes a living male, born in 1946 in Czechoslovakia, and immigrated to Israel in 1949. In 1978, he was diagnosed with testicular undifferentiated malignant teratoma (i.e., embryonal carcinoma). He was treated by surgery followed by chemotherapy. In 2007, he experienced a urinary bladder tumor, treated by surgery and followed by instillations. In 2016, he faced a metastatic papillary cancer of the thyroid, treated by surgery and radioactive iodine. It is noteworthy that his parents lived in Czechoslovakia under the strict restrictions of the Nazi German occupation from 1941 to August 1944, when they were transported to the Sered’ concentration camp in Slovakia. On April 1945, they were transferred to Theresienstadt concentration camp (Czech), from which they were liberated on 8 May 1945, the official date of the end of World War II. They returned to their home town and found out that his mother was pregnant. He was born after a difficult pregnancy, 286 days or 40.9 weeks after the liberation from the concentration camp. Therefore, the fertilization process was probably around liberation day.

## 4. In Utero Conditions and Cancer

The intra-uterine conditions during pregnancy—in utero—have been hypothesized for decades as part of the risk for development of cancer at any age between childhood and aged adults [24]. This hypothesis assumes that development of malignant cells may last for decades, and thus, the patient is exposed over this period to a variety of conditions starting from embryogenesis during maternal pregnancy. It is believed that testicular cancer is initiated from primordial germ cells or gonocytes that undergo improper differentiation during embryogenesis in the first trimester of pregnancy. During pregnancy, intrauterine estrogen concentration is increased approximately 10 times in the female and 100 times in the male fetus [25]. In utero increased exposure to estrogens, either endogenous or exogenous, in mice has been suggested to induce improper development of germ cells [26]. In humans, it has been shown that higher levels of neonatal androgens were associated with increased risk of TGCC among adolescents [27]. Twin pregnancies are characterized by high levels of hormones, including progesterone, and thus, the risk for testicular cancer is higher [25,28].

The placenta is the most important organ during pregnancy, in that it is developing concomitantly with the fetus. It is the fetus’ only source for nutrition and gas exchange with the environment and undergoes adaptation with the fetus’ needs. Extreme in utero conditions during embryogenesis in early pregnancy may damage the placental homeostatic capacity, resulting in oxidative and nutritive stresses with potentially adverse consequences for the fetus, including postnatal and later-in-life diseases [29,30]. While the literature is rich with human and animal reports and speculations, the mechanisms at the placental level are quite elusive.

**Figure 1:**
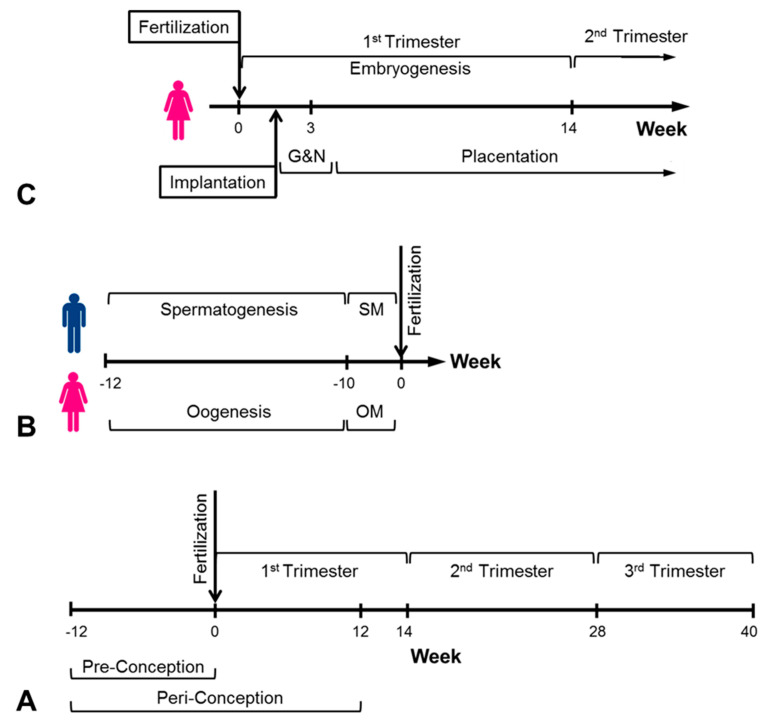
Schematic description of critical time periods during the human reproductive cycle. (**A**) Complete reproductive cycle; (**B**) The pre-conception period (SM—sperm maturation, OM—oocyte maturation); (**C**) Embryogenesis and placentation (G&N—gastrulation and neurulation).

A popular scientific hypothesis for in utero programming of later-life diseases is maternal malnutrition during pregnancy. The phrases nutrition programming and metabolism programming are used to describe the developmental origins of health and disease hypotheses for under-nutrition during the critical time window of early fetal development (Figure 1) that may lead to risk factors for non-communicable diseases [31,32,33]. However, the existing knowledge is mostly from small animal studies [34], while the few initial research efforts with humans are beyond the first trimester, when organogenesis takes place. The most famous relevant events are the Leningrad siege and Dutch famine during World War II [35,36]. While epidemiological analysis of data from survivors that were conceived during the Dutch famine demonstrated correlations with hypertension, diabetes and cardiac diseases, those who survived the Leningrad siege failed to demonstrate a similar trend. Nevertheless, hunger in the womb is most critical during early gestation, which is the most vulnerable period during fetal development [37]. It is interesting that these studies revealed that the adverse effects seem to depend on the timing during gestation and not on size of the baby at birth. Similar outcomes were also observed in undernourished populations in Africa.

In recent years, epigenetic mechanisms that program the individual risks for diseases in later life have been suggested to explain altered fetal development during early life [38]. Most of the epigenetic knowledge has been accumulated in animal studies that included modifications of DNA methylation, histone and non-coding RNAs that altered gene regulation without changing the DNA sequence. In humans, epigenetic analysis of blood samples from individuals who were prenatally exposed to hunger during the Dutch famine revealed changes in DNA methylation 6 decades later, as compared to unexposed controls [39,40]. However, it seems that most studies were based on trial and error experiments that demonstrated changes, but did not prove the developmental biology of specific diseases. The limited studies with humans were mostly focused on obesity, diabetes and cardiovascular diseases. The in utero or pre-conception origin of the epigenetic role for later-in-life diseases is still a matter of speculation, but the pattern of DNA methylation seems to be a major influential marker [40]. This is especially relevant to TGCC because the normal development of germ cells is subject to extensive epigenetic programming, and thus, improper DNA methylation is a prominent feature of tumor development [41,42].

## 5. Discussion

The origin of TGCCs that contain embryonic tissue is most likely established during fetal development due to an improper in utero environment. The time window of early human life (Figure 1) is composed of critical events in developmental biology, starting from the formation of the gamete cells up to the formation of a human-like fetus post-embryogenesis [43,44]. For example, in vitro nutritional manipulation during blastocyst formation in humans (i.e., about 5 days after fertilization) resulted in cleavage anomalies, while similar animal studies revealed a range of improper development and birth defects [45]. Moreover, epigenetic reprogramming during embryogenesis determines the expression of genes and imprinted genes that dictate cell phenotype and the fate of fetal development.

In recent years, the peri-conception window (Figure 1) has been defined as the period before (e.g., 3 months) and immediately after (e.g., 3 months) the time of conception [45,46]. This period includes maturation of oocytes and sperm, fertilization, blastocyst development, implantation, placentation and embryogenesis, which set up the critical foundations of fetal development. Scientists have realized that irregular or unnatural early events, including assisted reproductive technologies (ART), may interfere with the normal fetal development to ensure health and wellness in later life. Accordingly, the American College of Obstetricians and Gynecologists and the Centers for Disease Control and Prevention advised pregnancy planning couples to adopt healthy lifestyles during the peri-conception window and especially during pre-conception [47].

The actual linkage between TGCC and any specific improper in utero environment has not been demonstrated as it has been in all other non-communicable childhood or adulthood diseases. Nevertheless, the examples described earlier, in which extreme environments pre- and during conception led to TGCC in young adults, provide a logical linkage to improper in utero programming due to stressed environments. In the first example of the quadruplets, it was obvious that at least some of the fetuses were under-nourished and the probability of altered development was there. Of course, we do not have postnatal morphological and clinical details on these brothers, but the logical linkage to in utero conditions is obvious. From the perspective of obstetrics, one would assume that the diseased brothers were monozygotic twins that inherited defective epigenetic factors. It should be noted that the rare successful quadruplet pregnancies, whether spontaneous or post ART intervention, ended with preterm labor under week 32 with deficient development of the fetuses [48,49].

The second example of immediate post World War II pregnancy presented an extreme situation where the parents were held in vicious conditions for several years before and during pre-conception. Since men and women were held in separate sections in Theresienstadt concentration camp, it seems that conception occurred immediately after liberation of the parents. The mental and biological conditions of these parents were against all odds of the pre-parenting advisories [47]. Moreover, the harsh conditions of this pregnancy of a concentration camp survivor immediately post-war were extreme. The fact that multiple cancerous tumors in the offspring started with TGCC at a young age may suggest that its genesis was imprinted during in utero fetal development.

Many studies and reviews on the in utero origin of diseases focused on the effect of mal-nutrition. More recently published reviews emphasized the role of gonocytes, gene networks and DNA methylation in the development of TGCC and their in utero origin [50,51,52]. The physical in utero environment should also be added since the uterus is a dynamic organ due to spontaneous contractions of the uterine myometrium. In the non-pregnant uterus, the spontaneous contractions of the myometrial smooth muscle cells are regulated by the ovary and uterine hormones and play important roles during early human life [43,53]. After embryo implantation in the uterine wall, the uterus becomes passive until before initiation of labor while growing and stretching to support the expanding amniotic sac. During embryogenesis, mechanical forces derived from fluid flow stresses play crucial roles in the organogenesis of the heart, lungs, kidneys, brain and blood vessels [54,55,56]. Generation of improper distributions of forces is thought to be the origin factor for development of diseases during adulthood [7,57].

The existing knowledge of in utero programming of diseases has been accumulated from animal studies, usually with mice models due to ethical and accessibility limitations with humans. However, the outcome of animal models is not always applicable to humans due to differences between mammalian species [58]. Presently, tissue engineered in vitro models of living tissues and physiological patterns have been advanced to technological levels that support the potential for studying biological developmental processes without ethical and accessibility limitations [59]. For example, in a recent study, we demonstrated that quercetin, which is an abundant flavonoid in human diet and available in high concentrations in food supplements, can easily be transported through a tissue engineered placental barrier and reach the fetal blood compartment [60]. At the levels it reaches in the fetal blood, quercetin suppressed major DNA repair pathways and downregulated critical DNA repair factors in hematopoietic stem and progenitor cells. This suggested that high concentrations of quercetin in utero may damage the fetal DNA and result in the development of infant or adult leukemia. Similarly, future models of human primordial germ cell-like cells that can be cultured from human pluripotent stem cells [61,62] may serve to study the effect of sex steroid hormones at critical periods of development.

In conclusion, it is presently becoming the consensus view that extreme or irregular in utero environments, especially during the very early stages of the peri-conception period, program fetal development with increased risks for the outbreak of pathologies later in life. An obvious example is TGCC in adolescents and young adults. The origins of extreme in utero environments are mostly maternal-induced and may disturb the optimal molecular and biophysical in utero conditions. Moreover, precursors for TGCC may originate as early as during fertilization or implantation of the blastocyst, but issues of ethics and accessibility limit significant progress. Therefore, new, out-of-the-box tissue-engineered models of human developmental biology should be designed to expand the scientific basis for in utero programming of later-life disease development.

## Data Availability

This review is based on the published articles listed in the References. No additional data resources.
